# Nontuberculous Mycobacterial Disease Mortality in the United States, 1999–2010: A Population-Based Comparative Study

**DOI:** 10.1371/journal.pone.0091879

**Published:** 2014-03-14

**Authors:** Mehdi Mirsaeidi, Roberto F. Machado, Joe G. N. Garcia, Dean E. Schraufnagel

**Affiliations:** 1 Division of Pulmonary and Critical Care, University of Illinois at Chicago, Chicago, Illinois, United States of America; 2 Department of Medicine, University of Arizona, Tucson, Arizona, United States of America; Hopital Raymond Poincare - Universite Versailles St. Quentin, France

## Abstract

**Background:**

Environmental nontuberculous mycobacteria (NTM) are ubiquitous organisms with which humans commonly interact. The epidemiologic characteristics of NTM diseases including mortality rate and its associated factors remain largely unknown. In this study, we explored the geographical area of exposure and mortality and comorbid conditions of affected persons to determine environment, host, and host-pathogen interactive factors.

**Methods:**

We analyzed mortality related to nontuberculous mycobacterial infections from 1999 through 2010 by examining multiple-cause-of-death data from the National Center for Health Statistics. Among those who died with these diseases, we analyzed age-adjusted mortality rates, trends, associations with demographic variables, and comorbid conditions and correlated this information with similar data for tuberculosis-related mortality during the same time.

**Measurements and Mean Results:**

From 1999 through 2010, nontuberculous mycobacterial disease was reported as an immediate cause of death in 2,990 people in the United States with a combined overall mean age-adjusted mortality rate of 0.1 per 100,000 person-years. A significant increase in the number of NTM related deaths was seen from 1999 through 2010 (R^2^ = 0.72, p<0.0001), but it was not significant after adjustment for age. Persons aged 55 years and older, women, those living in Hawaii and Louisiana, and those of non-Hispanic, white ethnicity had higher mortality rates. Compared to tuberculosis-related mortality, chronic obstructive pulmonary disease, bronchiectasis, HIV, interstitial lung diseases, and tobacco use were significantly more common in persons with nontuberculous mycobacteria-related deaths.

**Conclusions:**

Nontuberculous mycobacteria-related death numbers are rising and are unevenly distributed. The strong association of nontuberculous mycobacterial disease with age suggests that its prevalence will increase as the United States population ages.

## Introduction

Environmental nontuberculous mycobacteria (NTM) are ubiquitous in nature and have been isolated from domestic and public water supplies, workplaces, and hospitals at rates with significant variations depending on the regions. [1] The organisms also vary widely in their ability to cause disease. This variation has been long suspected to be caused by environmental factors, but as infection rates have increased with no obvious environmental source host factors are believed to be more and more important in pathogenesis of NTM infections. More specifically, host-organism interaction can be caused by a specific type of environmental exposure, such as contact with a respirable aerosol or from a specific host factor, such as stagnant mucus in a bronchiectatic airway. [2], [3].

The increase in NTM infections in recent years is remarkable. The prevalence of diseases caused by these organisms was reported to be between 1.6 to 1.8 per 100,000 population in the 1980s, but recent North American studies have reported a higher prevalence of 14.1 per 100,000. [4] Studies also indicate that pulmonary infections caused by NTM are increasing in North America, especially in people over 50 years of age. [5], [6], [7] In these North American settings, the burden of nontuberculous mycobacterial disease exceeds that of tuberculosis. [8] The basis for this increase is unclear. Some speculate that improvements in diagnostics secondary to the development and widespread utilization of diagnostic support tools like computerized tomography (CT) scans or new laboratory methods has led to the identification of more cases that would have otherwise been misdiagnosed or overlooked. Another possibility could be less “herd immunity” because of decreasing rates of tuberculosis and less use of the Bacille Calmette-Guérin (BCG) vaccine. [9], [10] Also, the increase in number of immunocompromised hosts is suggested to be another contributing factor. [11] It is also assumed that the rise in NTM infections is congruent with the worldwide Human immunodeficiency virus (HIV) epidemic as well as scientific advancements leading to the more widespread use of chemotherapy and organ and tissue transplantation. [12], [13].

Epidemiologic factors of NTM diseases are particularly important in diagnosis and management when compared to other diseases. NTM diseases frequently present at various points of disease severity ranging from asymptomatic to critical and at risk of death. In many cases, demographic, geographic, and comorbid host factors may determine where one falls within this range.This study was designed to determine the trend of NTM-related mortality in the last decade and related comorbidities. We also aimed to discover current national and state prevalence and mortality rates and to learn the demographic, geographic, and comorbid conditions compared with persons suffering from tuberculosis. Understanding these relationships could help to define high-risk groups for death and develop strategies for targeting prevention, treatment, and treatment intensity.

## Materials and Methods

This study was reviewed and approved by Institutional Review Board of University of Illinois at Chicago (approval number of 2013–0446).

### Study Design and Patient Data

This was a retrospective, population-based comparative study of multiple causes of death data from the death certificates in USA between 1999 and 2010. The data was obtained from the Wide-ranging Online Data for Epidemiologic Research (WONDER) prepared by the Centers for Disease Control and Prevention (CDC). [14], [15].

The death certificates provided information on characteristics of the decedent, including age at death, gender, race, and conditions that led to death. Death certificates show a single underlying cause of death (immediate cause of death), up to twenty comorbidities, and demographic information. Diseases and related conditions reported on the death certificate are coded in accordance with the International Classification of Diseases, 10th Revisions (ICD-10) for 1999–2010 data. [16].

### Study’s Variables

Variables included in the analysis were age, gender, race-ethnicity, year of death, urbanization, place of death, cause of death, and comorbid conditions. Race-ethnicity was categorized according to US census standards as non-Hispanic white, Hispanic, Asian-Pacific Islander, non-Hispanic black (black), and American Indian-Alaska Native (Native American). Age at death was grouped into the age groups less than 1, 1–4, 5–14, 15–24, 25–34, 35–44, 45–54, 55–64, 65–74, 75–84, and ≥85 years.

The place of death was categorized as the hospital, the decedent’s home, hospice, and nursing home-long term care facility. To find an association between the urbanization level of residence and NTM-related mortality, the National Center for Health Statistics (NCHS) urban-rural classification was applied. Urbanization was classified as 1) *large central metropolitan*, which are counties with ≥1 million population that have the entire population in a major city, or contain at least 250,000 residents of any main city, 2) *large fringe metro*, which are counties with ≥1 million residents but do not meet the central metropolitan criteria, 3) *medium metro*, which are counties with between 250,000 to 999,999 residents, 4) *small metro*, which are counties with less than 250,000 residents, 5) *micropolitan*, which are cities that do not meet the metro criteria, and 6) *noncore*, which are rural counties. [17], [18].

### Study Definitions

NTM-related mortality was defined as the immediate cause of death from pulmonary or extrapulmonary nontuberculous mycobacterial infection. This included all observations that assigned any of the ICD A31 codes as the immediate cause of death. Tuberculosis-related mortality was defined by an immediate cause of death with ICD-10 codes of A16 to A19.

### Statistical Analysis

Counts and percentages were examined as predictors using crude odds ratios and were tested by χ^2^ tests or, if applicable, exact tests. The crude mortality rate was not used in this study because of the potential misleading information resulting from comparing rates over the time in different age groups. Age-specific tuberculosis-related mortality rates and 95% confidence intervals (CIs) were calculated for each age group. Age-adjusted mortality rates were applied to measure relative mortality risk among groups and over time. The selected population for the computation of age-adjusted rates was “2000 U.S. standard”. [19].

Univariate linear regression analysis was performed to evaluate the mortality from nontuberculous mycobacteria- and tuberculosis-related trends from 1999–2010.

In order to examine comorbid conditions, we compared NTM-related deaths with tuberculosis-related deaths from 1999 through 2010 and computed crude odds ratio (OR) comparisons of selected comorbidities. Data were suppressed by NCHS when the data indicating fewer than ten persons to meet the principles for confidentiality limitations. Death rates were labeled as “unreliable” when the numerator was less than 21. All analyses were performed using SPSS version 17 (SPSS Inc.; Chicago, IL).

## Results

### Nontuberculous Mycobacterial- and Tuberculosis-related Deaths Rates and Their Trends

**Figure 1:**
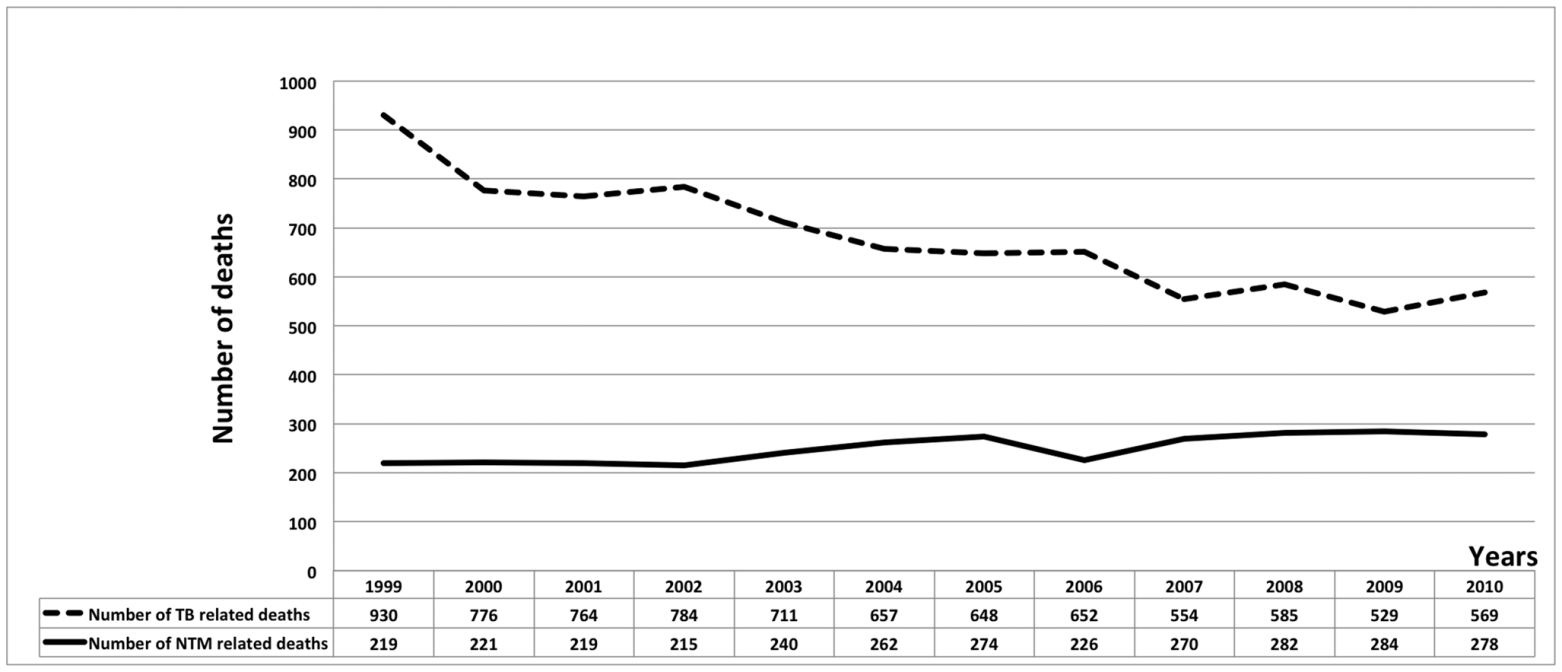
NTM and TB-related mortality rates per 100,000 person-years by year, United States, 1999–2010.

From 1999 through 2010, a total of 2990 immediate NTM-related deaths were identified, comprising 0.01% of the 29,176,040 total deaths in the United States. Comparing 1999–2004 to 2005–2010, the NTM age-adjusted mortality rates rose 10% (from 0.069 to 0.077 per 100,000 person-years). From 1999 through 2010, the number of NTM-related deaths rose significantly (R^2^ = 0.72, p<0.0001), but after correcting for the change in age distribution of the general population, this rise was not significant. Figure 1 shows the frequency of NTM and TB related deaths per 100,000 populations by year, in the United States from 1999–2010.

By comparison from 1999 to 2010, a total of 8159 immediate tuberculosis-related deaths were recorded. The number of TB-related deaths significantly decreased during this time interval (R^2^ = 0.94, P<0.0001), and TB-related age-adjusted mortality rate decreased as well (R^2^ = 0.92, P<0.0001).

### NTM-related Mortality Association with Age and Gender

**Figure 2:**
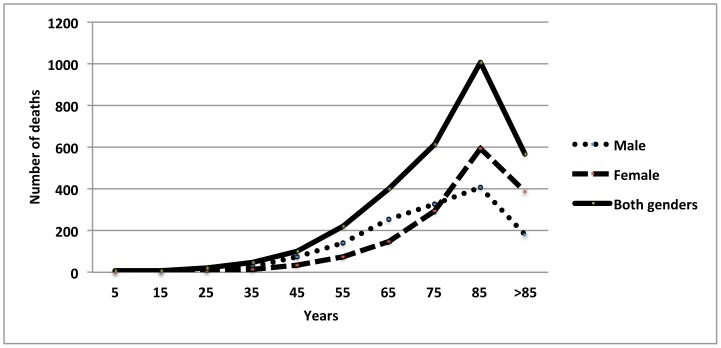
Number of NTM-related deaths stratified by age and gender United States, 1999–2010.

The majority (87%) of NTM-related deaths occurred in those older than 55 years of age. In comparison, 78% of tuberculosis-related deaths happened in persons older than 55 (P<0.0001) (see table 1 and Figure 2).

**Table 1:** Age-adjusted NTM and tuberculosis-related mortality rates per 100,000 person-years and Age-adjusted mortality rate ratios by gender, race/ethnicity, urbanization and age group, United States, 1999–2010.

Demographiccharacteristics	NTM		Tuberculosis	
	Number of deaths (%)	Age-adjusted mortalityrate (95% CI)*	Number of deaths (%)	Age-adjusted mortalityrate (95% CI)
**Gender**				
Male	1432 (48)	0.09 (0.08–0.09)	5005 (61)	0.32 (0.31–0.33)
Female	1558 (52)	0.07 (0.06–0.07)	3154 (39)	0.13 (0.13–0.14)
**Race**				
American Indian or Alaska Native	18 (6)	UR**	201(2)	0.92 (0.78–1.06)
Asian or Pacific Islander	112 (4)	0.09 (0.07–0.10)	1044 (13)	0.94 (0.88–1.00)
Black or African American	293 (10)	0.07 (0.07–0.08)	2067 (25)	0.57 (0.55–0.60)
White	2567 (86)	0.08 (0.07–0.08)	4847 (59)	0.15 (0.15–0.16)
**Hispanic**				
Hispanic or Latino	128 (4)	0.03 (0.02–0.03)	1179 (14)	0.44 (0.41–0.47)
Not Hispanic or Latino	2856 (96)	0.08 (0.07–0.08)	6911 (85)	0.21 (0.20–0.21)
**Urbanization**				
Large Central Metro	858 (29)	0.08 (0.07–0.08)	3373 (41)	0.32 (0.31–0.33)
Large Fringe Metro	593 (20)	0.07 (0.06–0.08)	1327 (16)	0.15 (0.14–0.16)
Medium Metro	709 (24)	0.08 (0.08–0.09)	1515 (19)	0.21 (0.20–0.22)
Small Metro	242 (8)	0.06 (0.05–0.07)	668 (8)	0.20 (0.18–0.21)
Micropolitan (non-metro)	376 (12)	0.10 (0.09–0.106)	721 (9)	0.17 (0.16–0.18)
NonCore (non-metro**)**	212 (7)	0.06 (0.05–0.06)	555 (7)	0.20 (0.19–0.22)
Age (in years)				
<1	3 (0.1)	UR	12 (0.2)	UR
1–4 years	4 (0.1)	UR	21 (0.3)	0.01 (0.01–0.02)
5–14	8 (0.3)	UR	17 (0.2)	UR
15–24	18 (0.6)	UR	106 (1)	0.02 (0.02–0.03)
25–34	46 (2)	0.01 (0.01–0.02)	214 (3)	0.04 (0.04–0.05)
35–44	102 (3)	0.02 (0.02–0.02)	452 (6)	0.09 (0.80–0.09)
45–54	219 (7)	0.04 (0.04–0.05)	1005 (12)	0.2 (0.19–0.21)
55–64	399 (13)	0.11 (0.10–0.12)	1176 (14)	0.33 (0.31–0.35)
65–74	617 (21)	0.27 (0.24–0.29)	1537 (19)	0.66 (0.63–0.70)
75–84	1006 (34)	0.65 (0.61–0.69)	2241 (27)	1.45 (1.39–1.51)
85+	568 (19)	1 (0.92–1.08)	1374 (17)	2.42 (2.29–2.55)
**Total**	2990 (100)	0.075	8159 (100)	0.22

* 95% CIs: 95% Confidence intervals,

**UR: Unreliable.

NTM-related mortality occurred in 1558 (52.1%) of women vs. 1432 (47.9%) of men.

Tuberculosis-related mortality occurred more in men than women, 5005 (61%) vs. 3154 (38%).

**Figure 3:**
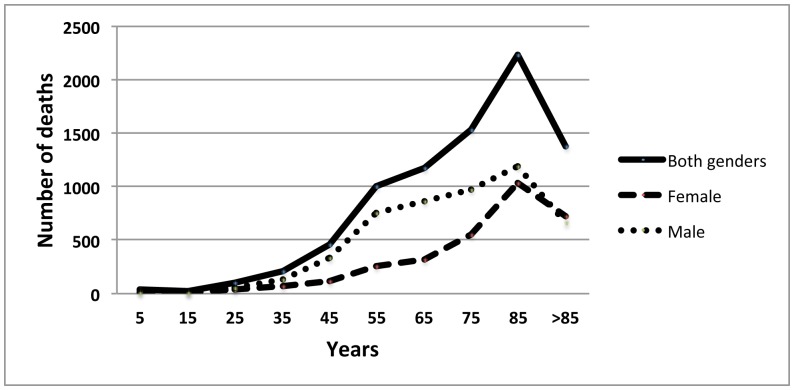
Number of tuberculosis-related deaths stratified by age and gender United States, 1999–2010.

Figure 3 shows the number of tuberculosis-related deaths stratified by age and gender.

### NTM and Tuberculosis-related Deaths and Ethnicity

The NTM related deaths rate between racial-ethnic groups was substantially different. Although the largest proportion of deaths occurred in whites (2567 deaths or 85% of all NTM-related deaths), they had a slightly lower age-adjusted mortality rate than Asian or Pacific Islanders (0.08 and 0.09 respectively). Tuberculosis-related deaths among ethnic groups were also disproportional. Among those with tuberculosis, 4847 (59%) were white, but the age-adjusted mortality was 0.15 person-years. It was lower than Asian or Pacific Islanders of whom1044 (12.8%) died with an age-adjusted rate mortality of 0.94 in 100,000 person-years (Table 1).

### NTM-related Deaths and Geographic Variation

**Figure 4:**
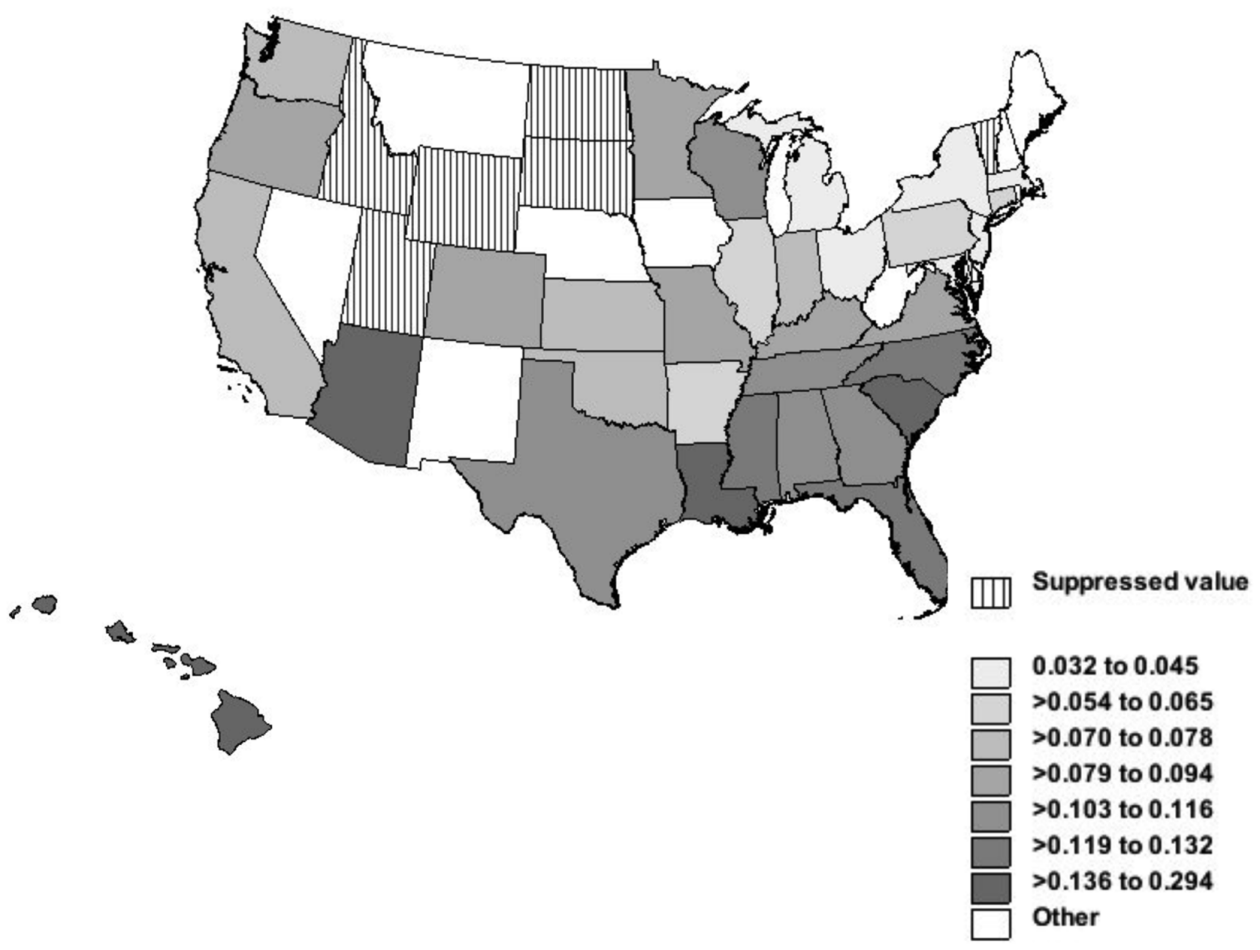
NTM-related deaths and age-adjusted mortality rates per 100,000 person-years by States, United States, 1999–2010. Legend: Alaska had NTM related mortality less than 9, therefore data were suppressed to meet the criteria for confidentiality constraints.

**Figure 5:**
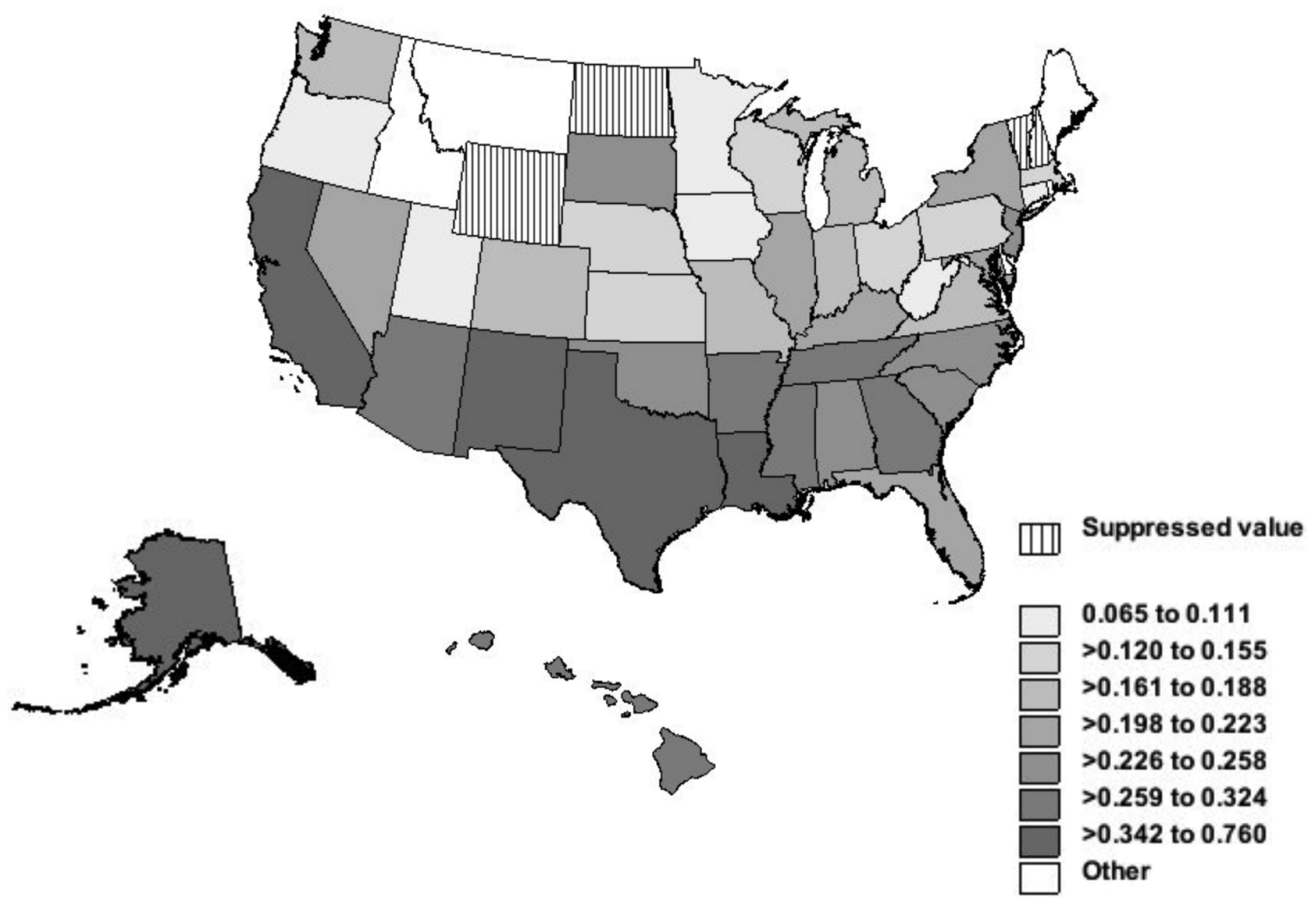
Tuberculosis-related deaths and age-adjusted mortality rates per 100,000 person-years by States, 1999–2010.

The NTM mortality rates varied among the states. The death rates for NTM-related disease and tuberculosis and age-adjusted mortality rates per 100,000 person-years are shown in figures 4 and 5. The age-adjusted NTM-related mortality rate was highest in Hawaii, 0.29 per 100,000 person-years. This was more than nine times higher than Michigan, which had the lowest rate of 0.03 per 100,000 person-years. Louisiana, Arizona, South Carolina, North Carolina and Florida had age-adjusted rates of 0.17, 0.14, 0.14, 0.13 and 0.12 per 100,000 person-years and came after Hawaii respectively (Figure 4). The tuberculosis-related mortality was strikingly different. Alaska had the highest age-adjusted rate of 0.76 per 100,000 person-years, which was 2.6 times more than the highest NTM-related age-adjusted rate (in Hawaii). After Alaska, the highest age-adjusted mortality rates for tuberculosis occurred in the District of Colombia, California, Texas, Louisiana, and New Mexico (Figure 5).

### NTM-related Mortality and Place of Death

When comparing NTM-related mortality by place of death, the majority of deaths occurred in hospitals (55%), nursing homes or long-term care facilities (12%), and in the decedents’ homes (23%). Among people who died from tuberculosis, significantly more individuals died in hospitals (76%) and fewer died in nursing homes or long term care facilities (7%), and at home (11%). Deaths occurred in hospital were significantly higher in TB group (OR = 2.2, P<0.0001).

### NTM-related Mortality and Urbanization

The comparison of NTM-related deaths with tuberculosis-related deaths by urbanization is shown in table 1. More than 51% of NTM-related deaths occurred in small and medium-sized metropolitan areas and only 29% occurred in large central metropolitan areas. However, the age-adjusted death rate related to NTM in the micropolitan group was 0.1 (CI 0.09–0.11), which is 1.3 times more than the age-adjusted NTM-related mortality in large central metropolitan group which was 0.08 (CI 0.07–0.08). There are no meaningful differences for NTM-related mortality associated with urbanization.

The tuberculosis-related mortality had a strong urban predominance; 41% of people dying with tuberculosis resided in large central metropolitan areas with age-adjusted mortality rate of 0.32 (CI 0.31–0.33). Only 8.8% of persons with tuberculosis died in micropolitan areas and had an age-adjusted mortality rate of 0.17 (CI 0.16–0.18) that was 1.9 times less than the mortality rate in large central metropolitan areas. There was a significant difference in mortality rate of large central metropolitan between NTM and TB groups (OR = 1.44, P<0.0001).

To investigate the reason for the differences, we hypothesized that access to a hospital might have been an important factor. We, therefore, compared the frequency of place of death in NTM-related deaths by urbanization categories. The percentage of persons who died in hospitals with NTM as the cause of death was 62% in large central metropolitan areas, 58% in large fringe metropolitan areas, 55% in medium metropolitan areas, 80% in small metropolitan areas, 52% in micropolitan areas, and 64% in non-core areas.

### NTM-related Deaths and Involved Organs

Among 2990 individuals who died with NTM diseases, 2413 (80.7%) had pulmonary NTM, 62 (2.1%) had extrapulmonary, and in 515 (17.2%) the organs involved were not specified. In the tuberculosis group, 6259 (76%) had pulmonary, 1179 (15.2%) had extrapulmonary, 669 (8.2%) had miliary tuberculosis, and in 52 (0.6%) subjects the organs involved were not specified.

Pulmonary involvement was reported significantly higher in NTM group (P<0.0001).

### Comorbidities

Compared with tuberculosis, several comorbidities were significantly more common in those dying from NTM diseases (table 2). COPD was reported in 24% of NTM group as comorbidity but only in 11% of TB group (OR = 2.5, P<0.001). However, the frequency of emphysema in two groups showed no significant difference. Bronchiectasis was significantly more common comorbidity registered in the patients who died from NTM diseases than tuberculosis (OR = 3.7, P<0.0001). Interstitial lung disease was found significantly higher in NTM compare to tuberculosis groups (OR = 1.63, P<0.0001). Conversely, Diabetes mellitus was significantly reported lower in NTM group than tuberculosis group (OR = 0.45, P<0.0001). HIV disease was reported significantly higher in NTM group as comorbidity than TB group (OR = 7.67, P<0.0001). The frequency of several other comorbidities showed no statistically significant difference in the NTM and tuberculosis groups including cystic fibrosis, sarcoidosis, heart failure, malignant neoplasm of trachea, bronchus and lung, malnutrition, systemic lupus erythematous, Crohn’s disease, any malignant neoplasm, alpha-1 antitrypsin deficiency, pneumoconiosis, deforming dorsopathies, and dementia including Alzheimer disease. The data were not shown.

**Table 2:** Frequency of selected comorbid causes of death and odds ratios (95% CI) comparing NTM-related mortality with tuberculosis-related mortality, United States, 1999–2010.

Comorbid condition	NTM (Total number: 2990)		Tuberculosis (Total number: 8159)		P value- OR** (95%CI)
	Frequency ofcomorbidconditions (%)	Age-adjustedmortalityrate (CI 95%)	Frequency ofcomorbid condition(%)	Age-adjustedmortalityrate (95% CI)*	
COPD	719 (24)	0.02 (0.02–0.02)	924 (11)	0.02 (0.02–0.02)	<0.01, 2.5 (2.2–2.8)
Chronic respiratory failure	39 (1)	TL***	47 (0.6)	TL	<0.01, 2.3 (1.5–3.5)
Mental and behavioraldisorder due touse tobacco	293 (10)	TL	373 (5)	0.01 (0.001–0.01)	<0.01, 2.3 (1.9–2.7)
Mental and behavioraldisorder due touse alcohol	15 (0.5)	UR^$^	117 (1.4)	TL	<0.01, 0.35 (0.2–0.6)
Bronchiectasis	244 (8)	0.01 (0.01–0.01)	192 (2)	0.01 (0.001–0.01)	<0.01, 3.7 (3–4.5)
HIV	14 (0.5)	UR	5 (0.1)	UR	<0.01, 7.7 (2.8–21.3)
Chronic renal failure	43 (1)	TL	242 (3)	TL	<0.01, 0.5 (0.3–0.7)
Liver disease	17 (0.6)	UR	148 (2)	NA	<0.01, 0.3 (0.2–0.5)
Diabetes Mellitus	102 (3)	TL	596 (7)	0.01 (0.01–0.01)	<0.01, 0.5 (0.4–0.6)
Interstitial lung diseases	196 (7)	0.01 (0.01–0.01)	336 (4)	0.01 (0.01–0.01)	<0.01, 1.6 (1.4–2)
Rheumatoid arthritis	51 (2)	TL	83 (1)	TL	<0.01, 1.7 (1.2–2.4)
Primary immunodeficiency	63 (2)	TL	44 (0.5)	TL	<0.01, 4 (2.7–5.9)
Lymphoma andhematopoieticmalignancies	32 (1.1)	TL	45 (0.6)	TL	<0.01, 2 (1.2–3)

* 95% CIs: 95% Confidence intervals, **OR: Odds ratio, ***TL: Too low when OR and 95% CI calculated as 0.001(0.001–0.001), ^$^UR: Unreliable.

## Discussion

This study showed that number of deaths from nontuberculous mycobacterial infection is increasing especially in older women. Hawaii demonstrated the highest age-adjusted mortality rate in the US followed by southern states in the west and east. Medium size metro and smaller areas have more than 50% of NTM related deaths. The majority of NTM related deaths occur in the hospital setting although this is less likely in small (micropolitan) cities. Although, we found no meaningful difference for NTM related mortality as was observed difference for TB related mortality on urbanization.

The NTM prevalence was 1.8 per 100,000 in the US during 1980s. [20] Winthrop et al. reported its prevalence was 8.6 per 100,000 in Oregon, USA in the 2005–2006, with an age-adjusted prevalence of 20.4 per 100,000 in those over 50 years old. In that cohort, the median age was 66 years and 59% were women. [5] A recent nationwide study in the US by Adjemian et al. showed the prevalence of NTM disease was increasing across all regions in the elderly. [21] Between 1997 and 2007, the annual prevalence increased from 20 to 47 cases/100,000 persons, or 8.2% per year among patients whom covered by Federal Health Insurance Program (Medicare beneficiaries). The period prevalence for this time was 112 cases/100,000 persons and was twice as much in Asians/Pacific Islanders as whites (228 vs. 116 cases/100,000 persons).

Our study also demonstrates the increase in numbers of NTM-related mortality from 1999 to 2010. We also show a striking association between NTM death and age, consistent with the analogous association between NTM prevalence and age. Given the increasing median age in North America, the prevalence and mortality of the mycobacterial disease will most probably increase. [22] In fact, the current increase in NTM disease in North America and the world could be explained, in part, by aging populations. [23].

Our study also points out the strong correlations between NTM-related mortality with many other medical conditions. The correlation with smoking, hematologic malignancy and HIV is stronger than with tuberculosis. As these chronic conditions increase, the risk of death from nontuberculous mycobacterial infection increases. It is important to note that many of the associated conditions involve mucus pooling in the airways or decrease clearance. The overwhelming percentage of disease being confined to the lung also favors an airway defense mechanism defect as a cause of NTM-disease.

Although tuberculosis-related deaths in the United States are sharply decreasing and NTM-related deaths are increasing, NTM-related mortality is much less than that of tuberculosis despite higher prevalence of the environmental mycobacterial disease. [6] One explanation for this discrepancy is that tuberculosis figures are more likely to be accurate due to public health reporting requirements. NTM diseases are not reported to public health departments which this has led many to speculate that NTM-disease is likely to be substantially underreported. Additionally, missed diagnoses in mild and asymptomatic NTM subjects may also decrease the number of the recorded cases. This occurs less likely for tuberculosis infections due to its uniformly virulent nature. [24] Finally, NTM may be a chronic and uncommonly fatal disease, with significant morbidity but relatively low mortality.

Although pathogen-host factors are important, there is geographical variation in NTM-related mortality. The southeast has long been known to have a higher prevalence of NTM, which may be attributed to its warmer and more humid climate. [25] Other studies have shown coastal area had greater number of cases, raising the conjecture that these mycobacteria flourished in wetter climates. [20] But our study showed that states with the drier weather, such as California, Arizona and New Mexico, also had high NTM-related mortality rates. Our findings are consistent by another NTM study. [21] Therefore, we believe that both warm and dry climates may contribute to NTM disease and influence on mortality by different environmental factors, such as temperature, soil and water conditions. Our study also suggests that living environment and possibly ethnicity contribute to NTM-related mortality. Our finding suggests that NTM related mortality is influenced much more by living in Hawaii than by being Asian/Pacific islander. Some of NTM-related deaths occurred in rural counties in our study. Rural communities may be associated with more contact to water and soil in rural agricultural settings [26] or less access to health care expertise, undermining the proper identification and management of NTM cases.

NTM demonstrated a strong relationship with some comorbid conditions such as chronic obstructive pulmonary disease, chronic respiratory failure, bronchiectasis, HIV, interstitial lung diseases, rheumatoid arthritis, hematopoietic malignancies and tobacco use. In comparison, tuberculosis is strongly associated with smoking, HIV and hematopoietic malignancies, and may cause bronchiectasis and chronic obstructive pulmonary disease. The association between aging and NTM might be explained by more prevalence of these chronic medical conditions in the elderly. In addition, the features of these conditions can play an important role in the pathogenesis of NTM diseases. For instance, bronchiectasis is a known lung abnormality that makes patient susceptible to NTM. [3], [27], [28], [29].

The main components of chronic obstructive pulmonary disease are emphysema and chronic bronchitis, although bronchiectasis is often present and unrecognized. [30] The association with stagnant mucus is established. The role of decreased airflow and cough pressure in the pathogenesis of NTM disease is unknown.

Primary immunodeficiency is another condition that was found in a few individuals in NTM-related deaths group. Although defense against mycobacterium invasion relies on innate and adaptive immunity [31], the small percentage of persons with known immune deficiencies diagnosed with NTM forces us to consider that structural defects of the epithelial barrier in addition to immune status may be a contributor. Bronchiectasis, for example, is associated with primary immunodeficiency and other conditions, such as rheumatoid arthritis. [32], [33].

The use of death certificate data is perhaps the most important limitation of this study. Death certificates are well known to misclassify variables. [34] In our study, we found that death certificate records were often unclear regarding whether the person died from mycobacterial disease or another cause of death. Due to the less severe disease presentation which is common in NTM, clinicians may not consider mycobacterial infections the immediate causes of death or an important underlying condition when completing death certificate. It is likely that physicians do not check the American Thoracic Society criteria for NTM disease before filling out the death certification. We believe that these poor documentations have a significant impact on NTM death certificates database. Unfortunately, death certificates database for NTM remains one of the more reliable data sources. Using ICD codes to detect nontuberculous mycobacterial disease may cause missing 25% to 75% of the cases. [35] Given the severe nature and clinical awareness for tuberculosis, it is unlikely that primary data in this arm is equally unrepresented. Physicians are more aware of tuberculosis because it is communicable, and reportable which requires health system monitoring. Therefore, it is much more likely to be reported accurately. Because environmental mycobacterial disease lacks these features, especially virulency, we think our data most likely represent an underestimate of true magnitude of the NTM mortality. Therefore, our findings might be interpreted as minimum estimates.

In summary, this United States population-based study shows that the number of deaths from nontuberculous mycobacterial disease is rising, but this increase is largely explained by the change in age distribution of the US population. There is considerable variation in different groups, which could be due to environment, host and host-pathogen interactive factors.
